# Review on Smart Gas Sensing Technology

**DOI:** 10.3390/s19173760

**Published:** 2019-08-30

**Authors:** Shaobin Feng, Fadi Farha, Qingjuan Li, Yueliang Wan, Yang Xu, Tao Zhang, Huansheng Ning

**Affiliations:** 1 School of Computer and Communication Engineering, University of Science and Technology Beijing, Beijing 100083, China; 2 Beijing Engineering Research Center for Cyberspace Data Analysis and Applications, Beijing 100083, China; 3 Research Institute, Run Technologies Co., Ltd. Beijing, Beijing 100192, China; 4 Key Lab of Information Network Security of Ministry of Public Security (The Third Research Institute of Ministry of Public Security), Shanghai 201204, China

**Keywords:** smart gas sensing, gas sensor, sensor arrays, machine learning, sensitive, selectivity

## Abstract

With the development of the Internet-of-Things (IoT) technology, the applications of gas sensors in the fields of smart homes, wearable devices, and smart mobile terminals have developed by leaps and bounds. In such complex sensing scenarios, the gas sensor shows the defects of cross sensitivity and low selectivity. Therefore, smart gas sensing methods have been proposed to address these issues by adding sensor arrays, signal processing, and machine learning techniques to traditional gas sensing technologies. This review introduces the reader to the overall framework of smart gas sensing technology, including three key points; gas sensor arrays made of different materials, signal processing for drift compensation and feature extraction, and gas pattern recognition including Support Vector Machine (SVM), Artificial Neural Network (ANN), and other techniques. The implementation, evaluation, and comparison of the proposed solutions in each step have been summarized covering most of the relevant recently published studies. This review also highlights the challenges facing smart gas sensing technology represented by repeatability and reusability, circuit integration and miniaturization, and real-time sensing. Besides, the proposed solutions, which show the future directions of smart gas sensing, are explored. Finally, the recommendations for smart gas sensing based on brain-like sensing are provided in this paper.

## 1. Introduction

**Figure 1:**
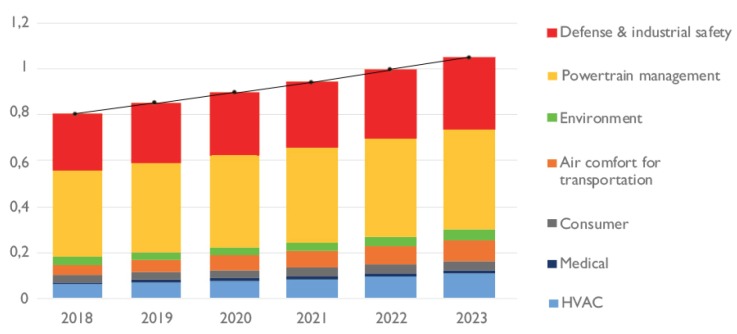
2018-2023 Gas Sensor Market in Value($B) [12].

In recent years, with the development of IoT technology, gas sensing has been firmly integrated with smart life and smart industry: (1) Food testing (e.g., predicting the freshness of pork, beef and mutton [1] and detecting the fresh vegetables freezing time [2]); (2) Animal and plant breeding (e.g., determine the time of cow estrus [3] and determining the time of fruit ripening [4]); (3) Air detecting (e.g., detecting ozone content [5] and air quality monitoring [5]); (4) Disease diagnosing (e.g., detecting liver cancer based on breath [6] and detecting gastrointestinal diseases [7]); (5) Industrial field (e.g., oil and gas positioning) [8] and pipeline leak detection [9]; (6) Hazard monitoring (e.g., explosion and toxic gas detection [10], fire warning [11]) etc. Although smart sensing has played a role in life and production, it should be emphasized that there are still many challenges in the development of smart gas sensing corresponding to the various stages of technology mentioned above. Yole Développement [12] draws a distribution map of the gas sensor market as shown in Figure 1. The gas sensor market is expected to grow at a rate of 6.25% per year and exceed 1$B in 2022 [12].

Unlike other sensing types, such as force sensing or temperature sensing which detect a single variable, the principle and the tasks of gas sensing are complex. Considering the sensing scenarios mentioned above, gas sensing encounters many challenges; (1) Cross sensitivity and weak selectivity: Gas sensors always detect gases depending on their chemical properties, e.g., redox properties. The gas sensor will be affected by non-target gases which have similar chemical characteristics while detecting a mixture of similar gases. For example, the cupric oxide (CuO) can be used in producing humidity sensors, but it is at the same time, sensitive to hydrogen (H_2_), ethanol (C_2_H_5_OH), nitrogen dioxide (CO), as well as hydrogen sulfide (H_2_S) [13]. (2) Low selectivity: The accuracy of the gas sensor is directly related to temperature and humidity [14]. Since gas activity is affected by temperature, the ability of the sensor to adsorb the gas varies at different temperatures even though most gas sensors often work well at high temperatures of several hundred degrees. On the other side, the humidity affects the interaction between the gas and the sensor, which makes it unpredictable. The sensor resistance is usually used as a measure of sensor response, which detects the current or voltage changes. The temperature drift caused by high temperatures often causes the resistance of the sensor to fluctuate up and down, and thus, affecting the measurement accuracy [15].

**Figure 2:**
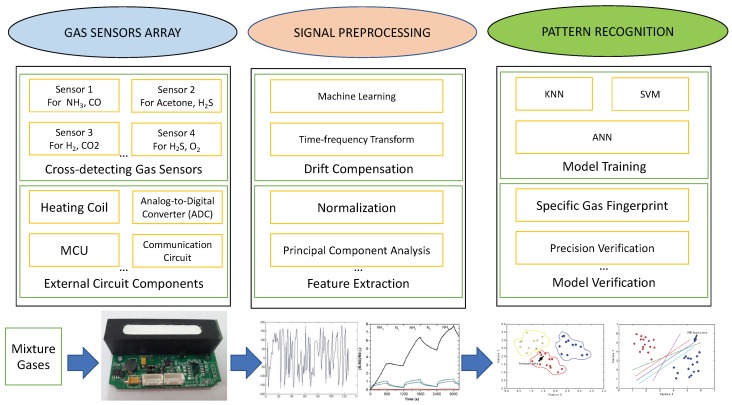
The step of Smart Gas Sensing.

In order to overcome the challenges as mentioned earlier, the concept of smart gas sensing has been proposed, as shown in Figure 2.

Smart gas sensing technology is a combination of a gas sensor array and pattern recognition method to detect, analyze, and quantify mixed gases, which can achieve high measurement accuracy and get smarter conclusions, such as judging the maturity according to the odor emitted from the fruit. Generally, sensors in the array response to the gas generating a unique set of signals called gas fingerprint, and then, characterizing various odors or volatile compounds by pattern recognition [16].

This review summarizes the relevant researches published in the last ten years to provide a comprehensive study on smart gas sensing technology. The remainder is organized as follows: Section 2 introduces gas sensors array and signal pre-processing. Section 3 presents gas pattern recognition. Section 4 lists the challenges facing smart gas sensing and the proposed solutions technology. Section 5 is a summary of smart gas inspection and its future development direction.

## 2. Gas Sensors Array and Signal Preprocessing

In the early 1980s, gas sensors arrays, which were made of many materials with different sensitivities and selectivities, were used and manufactured [17]. However, due to the limitations of low accuracy and drift phenomena, gas sensor array technology did not mature until nearly a decade. This section introduces various gas-sensitive materials, including principle, application scenario, advantages and disadvantages, and performance parameters.

### 2.1. Gas Sensitive Materials and Their Sensors Array

**Figure 3:**
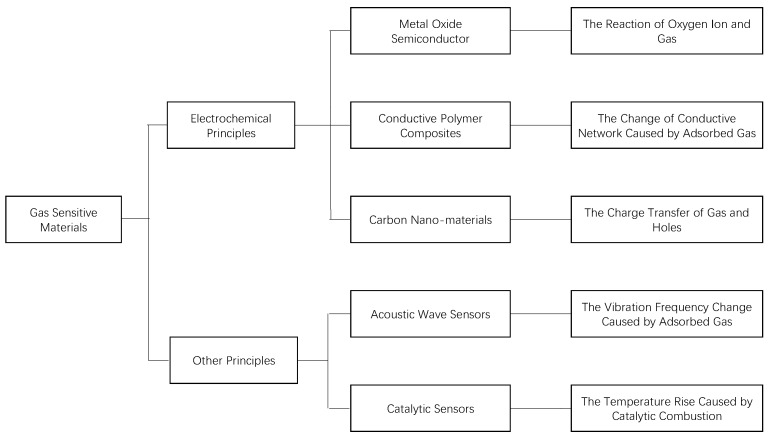
Classification of Gas Sensitive Materials [18,19,20,21,22].

According to the principle of gas sensing, gas-sensitive materials which are suitable for the sensor array are classified into two types based on electrochemical components and other principles, as shown in Figure 3.

There are three common materials used as sensing elements: metal oxide semiconductor (MOS) [23], conductive polymer composites (CPCs) [24], and carbon nano-materials [25].

**Figure 4:**
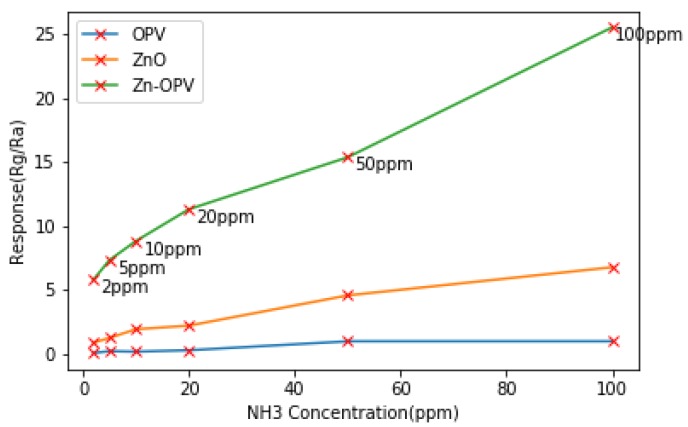
The Performance of Zn-OPV for Detecting NH${}_{3}$ at Room Temperature [33].

Common metal oxide semiconductor gas sensing materials include: NiO [26], SnO_2_ [27], Fe_2_O_3_ [28], ZnO [29], etc. Those materials are the most commonly used in manufacturing the gas sensors, especially for sensing an oxygen-containing gas. The major challenge for MOS is the high temperatures of the sensing operation, which causes temperature drift. The currently used solution is to adjust the structure of the metal oxide through nanotechnology and composite materials [30]. For structure solution, in the review of Li et al. [31], the design method and mechanism of detecting harmful gases by different nanostructured metal oxides at room temperature have been introduced. In another research, Oosthuizen et al. [32] presented using a hydrothermal-assisted method to synthesize CuO nano-platelets, which have highly sensitive and selective for detecting CO at room temperature. For composite solution, in the research of Aaryashree‘s et al. [33], ZnO and functionalized Oligophenylenevinylene (OPV) hardly detect Ammonia (NH${}_{3}$) at room temperature. While Zn-OPV composite, which is formed by the functionalized OPV interacting with the inorganic ZnO, shows a larger detection range and stronger sensitivity to NH${}_{3}$ than OPV or ZnO at room temperature as shown in Figure 4. Rg is the resistance in the presence of the target gas, and Ra is the resistance in the air or no target gas.

Conductive polymer composites (CPCs) are composite materials prepared by dispersing, mixing polymer material as a matrix, and a conductive filler as a filler [34], including polyaniline (PANI) [35], polypyrrole (PPy) [36], polythiophene (PTh) [37], and their derivatives. These components are essential gas-sensitive materials which operate well at room and low-temperatures. Due to the complex nature of organic matter, CPCs can be chemically modified using aromatic electrophilic substitution or nucleophile addition and then be deployed as active materials in resistive sensors [38]. For example, F. Miramirkhani et al. [39] compared four polymer gas sensing materials, including two conducting polymers (polypyrrole and polyaniline) and two polymer/carbon black (CB) composites consisting ethyl cellulose (EC) and polycaprolactone (PCL). The results show that polyaniline indicates the highest response to ammonia due to the effect of p-phenylene resonance on deprotonation process, and the PCL/CB sensor response is more pronounced compared with EC/CB as its higher porous structure.

Carbon nano-materials mainly include graphene and carbon nanotubes (CNTs). As a typical representative of two-dimensional nano-materials, graphene has a thickness of atomic size, every atom of graphene may be considered a surface atom and as a result, every atom site may be involved in the gas interactions, which causes its high sensitive sensor response with the lowest detection capability to even a single molecule [40]. Further, such graphene oxide (GO) [41] and reduced graphene oxide (RGO) [42] which are gained by functionalized graphene have been got more and more attention for their sensitive for different kinds of gases [43,44,45].

CNTs were firstly named in 1991 by Professor Iijima of NEC, Japan [46]. The structure of CNTs can be visualized as cylinders made up of rolled graphene layers. CNTs can be classified as single graphene wall (SWCNTs), or multiple walls concentrically arranged (MWCNTs) [47]. Strong adsorption, organic affinity, high specific surface area, flexibility, high sensitive, and functionalization of the surface make CNTs widely used for biomolecule detection and gas sensing [48,49,50]. Carbon nanotubes have high potential performance because of their large surface area, excellent electron transfer, and the ability to be manipulated with other conductive materials and polymers, in order to form chemically active sites for application purposes [51], including functional group modification [52], structural modification [53], and doping nano metal particles [54].

There is a class of mass-sensitive sensing materials to make acoustic wave sensors (e.g., Quartz Crystal Microbalance (QCM) [55] and Surface Acoustic Wave (SAW) [56]) which includes corresponding relationship between the vibration frequency and the quality. However, those materials have no direct physical or chemical reaction because of the target gas. [57] Usually, thin layers, which can adsorb the target gases, cover the frequency materials. Then, the gas properties can be measured by analyzing frequency changes of materials and characteristics of adsorbed materials [30]. QCM and SAW can be combined with an organic coating to achieve machine olfaction because of the extreme sensitivity of frequency changes [58]. Also, using such materials enables the humidity sensors to sense the water vapor by measuring changes in the adsorption mass rather than measuring the redox reactions [59,60,61,62]. Table 1 shows some humidity sensors based on QCM/SAW reported in recent years. In the Table, the "RH" means relative humidity in the air. The literature [59] shows average sensitivity, whereas the others show the sensitivity at a specific humidity.

Catalytic sensors are widely used in real-time leakage and online pipeline inspection [63] due to their low cost, sensitivity only to flammable gases and vapors, small size, and weight. Target gases are burned on the catalyst (the most common type is TiO${}_{2}$ [64]) and produce a specific combustion enthalpy enabling low-concentration analytes to be detected in short response times. However, it is difficult to measure the concentration of flammable mixed gases in the air. A. Somov et al. [65] propose a solution to this problem based on the measurement of heat dissipated during the mixture oxidation at a slow rate. A. Karelin et al. [66] propose an alternative way to measure the concentration of flammable mixed gases in the air when the flammable gas type is not known.

A summary of different gas-sensitive materials and methods is introduced in Table 2. There are also some gas detection methods based on different properties, such as optical methods [67], ultrasonic measurement method [68], gas chromatography [69], mass spectrometer [70], and spectrometer [71].

The challenges in the gas sensing field, such as low precision and cross-selectivity, can be solved by using sensor arrays based on different sensing technologies and different sensing materials. The main principle is that the sensor array generates different signal responses to different gases forming a unique gas fingerprint. For the gas fingerprint to be as easily identifiable, the choice of sensing material in the sensor array becomes critical. The commonly used method is based on the same substrate material mixed or coated with different materials, which is convenient to remove noise [72,73]. Also, some scholars have studied how to optimize choosing the sensors in a sensor array. Gustafson et al. [74] use the genetic algorithm to identify optimal combinations of metal-organic frameworks for detecting methane leaks in the air. The results show that the genetic algorithm can accurately predict the best arrays of any desired size when compared to brute-force screening. Ghasemi-Varnamkhasti et al. [75] optimize MOS array using response surface method (RSM), which considers the contribution of each sensor in a sample classification. Besides, Subandri et al. [76] provide another optimization idea with a contribution of the detecting gases rather than sensors. In their research, ten initial sensors are used in the e-nose prototype and then reduced to four sensors based on volatile organic compounds concentration data to minimize the use of sensors.

### 2.2. Drift Compensation and Feature Extraction

The upper limit of gas recognition accuracy is determined by the data feature. The original data have many problems, such as different dimensions, low information utilization, noise, and error. As a result, it is necessary to preprocess the data.

Drift in the sensor is a relatively complex and unavoidable phenomenon, which is produced in different sensing steps, such as aging and poisoning of the sensor, temperature and humidity from the environment, and the delay of data transmission [77]. Initially, the relevant researchers used uni-variate methods to compensate each sensor response individually by baseline manipulations: difference, relative, and fractional [16,78]. Although these approaches can partially handle drift, they are sensitive to sampling frequency and have poor robustness [79]. In multivariate methods, using information from multiple sensors has been proposed to capture more complex or non-linear drift effects in order to model the drift at the expense of increasing the number of parameters involved in the correction and frequent sampling [80,81].

The current exciting research in the field of drift compensation is using a machine learning method with self-adaptation that can effectively tackle the mismatches between real data and signal with drift. Besides, it does not need to recalibrate the sensor. There are several contributions available in the scientific literature that can solve sensors drift. S. De Vito et al. [82] reported using semi-supervised learning (SSL) to tackle the drift problem in a dynamic pattern recognition framework by adapting a regressor/classifier as unlabeled samples. Q. Liu et al. [83] reported using a domain adaptation algorithm based on the weighted geodesic flow combination kernel, selected unlabeled data, and semi-supervised method to deal with sensor drift. Even though domain adaptation can handle discrete source and target domains, it is not suitable for solving time-varying drift [84]. K. Yan et al. [85] proposed maximum independence domain adaptation (MIDA) and semi-supervised MIDA to address this problem.

To overcome the challenge of uniquely labeling the sensor devices in MIDA, some have proposed a discrete binary version of Particle Swarm Optimization [86] called DBPSO to search for drift insensitive features. In their experiment and the one carried out by [87], the results showed that the proposed algorithm is robust against the drift without requiring any re-calibration, domain transformation, or data from the target domain. In short, solving drift is a regression problem in machine learning. How to use the model summarized by existing data to correct the data with noise is still a challenge.

**Figure 5:**
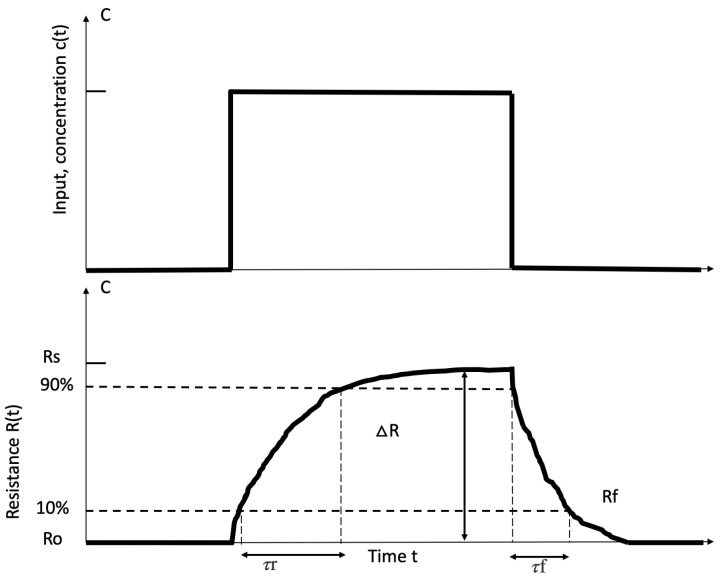
Typical Response of a Chemical Gas Sensor [89].

In addition to the drift compensation, another essential step is the feature extraction of the response from the sensor array. The purpose of feature extraction is to extract robust information with less redundancy from the feature sensor response, which can represent different “fingerprint” patterns and ensure the validity of subsequent pattern recognition algorithms. In Yan’s review [88], many feature extraction methods have been introduced in details, including original response curves, curve fitting parameters, transform domain, phase space, etc. Figure 5 [89] shows two standard parameters from baseline manipulation technique: ΔRs and ΔRf. Ro is the initial resistance of a sensor, Rs is the steady-state resistance, and Rf is the10% of response sensor at the end of the acquisition period. ΔRs means the resistance of sensor rises from Ro to Rs, which provides information about the response time *τ*s and ΔRf shows the resistance of sensor rises from Rf to Ro providing the recover time *τ*f.

It is still a considerable challenge to improve the pattern recognition accuracy by extracting more appropriate features from “fingerprints”. The most common methods used to extract features are normalization, Principal Component Analysis (PCA) [89] and Linear Discriminant Analysis (LDA) [90].

Normalization is used to correct over-characterized features extracted from different sensors, which may affect the analysis results and accelerate the speed of computational convergence. In Casey’s research [91], field normalization is useful means of quantifying gas mole fractions from raw sensor signals, which resolves the response of the sensor that results from other chemical and physical processes of the environment. Tonezzer et al. [92] compare the effects of different normalization methods on visual gas detection systems and qualitatively distinguish different concentrations of the same gas by normalizing the subset of data.

**Figure 6:**
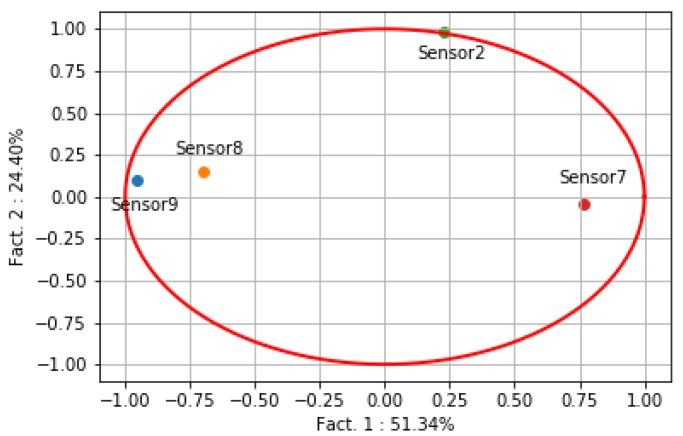
PCA Result of Each Sensor of the Array [95].

PCA plays an important role in enhancing gas sensor selectivity, which reduces the signal dimension from hundreds of wavelengths within the detection range to only the primary component that produces the most useful information while preserving multiple sensing mechanisms of different wavelengths [93]. Konstantynovski et al. [94] use PCA to process signal change rates from physical sensors and corrected resistance value from metal oxide gas sensors. R. Faleh et al. [95] use a sensor array based on WO${}_{3}$ gas sensors to detect O${}_{3}$. In their research, PCA is used to evaluate the contribution to the classification in order to enhance the sensors’ selectivity based on a database with four gas sensors. The advantage of PCA is to eliminate the correlation between evaluation indicators, reduce the workload of indicator selection, and keep most of the information while saving data space. As shown in Figure 6 [95], fact 1 (the response time parameter) and fact 2 (the separation concentration) use only 75% of the information to represent 99% of the information of gas database.

LDA is also widely employed to process the sensor array signal to get representative features. Different from PCA, LDA is a supervised dimensionality reduction algorithm, which uses reasonable label information to make the dimension discriminative, thus maximizing the separation of different categories of data. Wang et al. [96] use fisher LDA (FLDA) to identify if the duck meat is mixed in the mutton. For qualitative analysis, they just use linear regression and FLDA to finish the work. For quantitative analysis, LDA is just used to extract relevant features from the data. Ma et al. [97] use LDA to extract 33 elemental features from 313 tea samples from representative provinces in China, which achieved high recognition and satisfactory predictive ability. Liu et al. [98] have proposed the technique of extracting and detecting athletes’ oral odors based on the analysis of biological characteristics. The results show that LDA is superior to PCA in classifying patients with diseases and healthy people. This good performance of LDA is due to the known label data. But if feature extraction or dimensionality reduction is needed without known data, PCA will be a better solution.

## 3. Gas Sensing Pattern Recognition

Pattern recognition technology is a fundamental technology of artificial intelligence which has been widely used in many science technology fields, involving information detection, feature analysis, data processing, automatic controlling and so on.

Gas pattern recognition infers the unknown gas based on their characteristics by data sampling quantification, analysis processing, feature extraction, and classification decision [99]. Smart gas sensing builds multidimensional data by gas sensor arrays to improve recognition accuracy.

Pattern recognition technology is mainly divided into two categories, linear classification based on statistical theory and nonlinear classification based on neural network. Its application consists of two processes, training learning, and test evaluation. Training learning builds the rules of the decision model; test evaluation tests the accuracy and performance of the model.

### 3.1. Linear Classification Based on Statistical Theory

Traditional linear classifications based on statistical theory include K-Nearest Neighbor (KNN) and Support Vector Machine (SVM), which can train crossed, overlapping, and high dimensional samples in gas pattern recognition.

It is easy to build a training model for KNN: find the closest k training samples to the forecast data from the training set, and the forecast data is classified into the maximum probability classes among the k training samples [100]. Euclidean distance or Manhattan distance is common used to calculate similarity of training sample X = {$X_{1}$, $X_{2}$, $X_{3}$... $X_{n}$} to forecast data x = {$x_{1}$, $x_{2}$, $x_{3}$... $x_{n}$}.

KNN [101,102,103,104,105,106,107] is widely used for the classification of mixed gases due to its simplicity. Deng et al. [101] proposed a new feature selection based on KNN to enhance the discrimination ability of gas sensor arrays for odor identification and, to a certain extent, suppress drift effect. Experimental results on two datasets show that the recognition rates of Database I and Database II achieve 97.5% and 80.11%. Mawardzi et al. [102] use KNN intelligent classification technique to classify the four types of waxy crude oil with zero percentage of error. Ali et al. [103] designed fast prototyping of KNN based gas discrimination system. The best results are obtained for k = 1 and k = 2 with a classification accuracy of 97.91% and 98.95%, respectively. The value of k is the key to the KNN Model, which would be over-fitting and unstable if k is small. While k is large, the model would be under-fitting, and the result would always be classified to the class with the largest proportion of the training set. Usually, the most appropriate k value is chosen by a cross-validation method [104]. As lazy learning, disadvantages of KNN are that the training data must be pre-stored, having massive investment, and strong ability of processing to classify the new pattern. Brahim-Belhaouari et al. [105] have presented Cluster-K-Nearest Neighbors (CKNN) and tree-CKNN to overcome these drawbacks by simplifying the density estimation. Yang et al. [106] have introduced a novel clustering-KNN rule for real-time monitoring of gas sensor arrays with a large volume of variables and training samples. Yu et al. [107] have proposed a random subspace ensemble framework based on HBKNN (RS-HBKNN) classifier to perform classification on the datasets with noisy attributes in the high-dimensional space, which solves the problem that KNN is sensitive to noise on datasets.

SVM is a two-class classification model which is defined as the most significant distance linear classifier on the feature space. The primary linear classifier is to find a hyperplane in the sample space to separate the samples of different categories. SVM is a binary classifier for two-category samples by the maximum-margin hyperplane. This method has a good performance to generalize the unknown instance, which means good robustness. However, the gas characteristic information is often complicated, which is a non-linear relationship and cannot find the hyperplane to classify. The kernel function is a typical map method used to solve the non-linear problem in SVM, which linearizes nonlinear data by mapping to high dimension expression space [108,109].

SVM [110,111,112,113,114,115] attracts considerable attention in gas classification due to its high performance towards small samples and nonlinearity problems of the dataset. Jia et al. [111] present a method for gas-composition-unknown recognition by analyzing gas acoustic relaxation absorption spectrum based on wavelet multi-resolution analysis and multi-class SVM. Sujono et al. [112] design an asthma identification system by gas sensors and SVM. In their research, the system has high sensitivity, specificity, and accuracy in distinguishing between healthy and asthma subjects but low accuracy to distinguish the subjects of asthma with varying severity. To improve the prediction accuracy of SVM, Wang et al. [110] use a Genetic Algorithm (GA) to estimate the most suitable training parameters for SVM by assigning the training model parameters of SVM as its chromosome. In research of Zhang et al. [113], Particle Swarm Optimization (PSO) and GA, are used to optimize the SVM model parameters for improving the estimation accuracy of atmospheric NH${}_{3}$ concentration levels. The result shows that PSO-SVM provides higher retrieval accuracy and faster running speed than GA-SVM.

SVM has excellent use prospects in improving the accuracy of the sensor. A. Vergara and S. Vembu [114] collected an extensive dataset for six different volatile organic compounds over three years under tightly controlled operating conditions using an array of 16 metal-oxide gas sensors. The SVM of classifiers can cope well with sensor drift and perform better than the baseline competing methods. Laref et al. [115] calibrate the accuracy of the electronic nose dedicated to monitoring nitrogen dioxide by SVM and get good results compared to traditional calibration.

### 3.2. Nonlinear Classification Based on Artificial Neural Networks

Artificial Neural Networks (ANN) has been a research hotspot, aiming to understand the parallel computer system of neurons (the concept of flexible connection), imitating the human brain to solve various practical problems [116]. With the advent of complete theory and enhanced computing power, ANN begins to be applied to gas sensing in all kinds of complicated environments [91].

The neuron model is the most fundamental component of ANN, and the McCulloch–Pitts Model [117] shows how neurons work in ANN, every neuron receives and sums the signal from the previous layer which has the connection with weight, compares the weighted sum with threshold value, and generates outputs by appropriate activation function (e.g., Sigmoid function).

The general neural network is a multi-layer structure, which is divided into an input layer, hidden layer, and output layer. The hidden layer and the output layer are functional neurons having an activation function. The learning process of the neural network is to adjust the connection weight between the neurons and the threshold of each functional neuron according to the training data.

In general, the prediction accuracy of an ANN is in direct proportion to the number of layers in a hidden layer [91], but too many hidden layers will lead to the model overfitting. The error backpropagation (BP) algorithm has been proposed to solve this problem [118]. The main principle of BP is to adjust network weights and thresholds through error feedback [119,120]. The BP algorithm requirs a long training time. So a fast neural network algorithm named extreme learning machine (ELM) has been proposed [121]. In L. Zhao’s research [122], three recognition methods, including BP neural network, support vector machines (SVM) and extreme learning machine (ELM) with PCA, are compared for formaldehyde detection in mixed VOCs gases. The results show that ELM is fast, while the SVM performs with the best accuracy, as shown in Table 3, ‘W’ stands for the wrong prediction and ‘C’ stands for correct prediction.

ANN [91,119,120,121,122,123,124,125,126] is a universal method for prediction and analysis of complex gas which sufficient approximates the nonlinear relation and has the ability of associative learning. Casey et al. [91] use low-cost sensors to quantify 4 trace gas species in an oil and gas production region, and confirm ANN can represent the complex nonlinear behavior in response to the ambient environment among multiple gas sensors, including temperature, humidity, and atmospheric chemistry. Cheng et al. [123] use ANN to study the effect of steam flow rate (SFR) and reaction temperature on gas yield (GSY) and hydrogen yield (HDY). Ye et al. [124] use ANN to simulate methanol production from fuel gas derived from steam reforming of natural gas. Gao et al. [125] use ANN to study the influence of equivalence ratio (ER) on gas distribution, lower caloric value (LHV) of producer gas, and performance indicators (char conversion and cold gas efficiencies). ANN also can be used for the Machine Olfaction with the development potential. Prediction of odour intensity, the hedonic feel of human or odour interactions in multi-component mixtures is a difficult task, which is an application of non-linear methods. These requirements can be fulfilled by ANN with a sufficient number of hidden layers and suitable activation functions [126]. For example, in Aleixandre’s [127] and P. Qi’s [128] work, ANN is confirmed to mimic the taste of humans for food tasting, which can distinguish the taste of different red wines and Chinese liquors.

A comparison of the algorithms mentioned above has been given in Table 4. Each algorithm performs good in its field, and sometimes it can be combined with other algorithms to solve difficult problems.

## 4. Challenge of Smart Gas Sensing and Their Solutions

The technical framework for smart gas sensing has matured, including sensor arrays and pattern recognition technology. However, it should be noted that most of the smart gas sensing technologies are still under research and have not been applied widely. Mainly due to there are still many challenges in the development of smart gas sensing, which correspond to the various stages of technology mentioned above. The problems of reliable sensor reusability and large-area sensing field have not been well solved. In addition, how to ensure the established gas model can be quickly applied to different sensing scenes is still a problem. In the IoT scenario, a weakly compute-able terminal quickly processes a large amount of sensor array data is another gap between hardware and software.

### 4.1. Repeatability and Reusability

**Figure 7:**
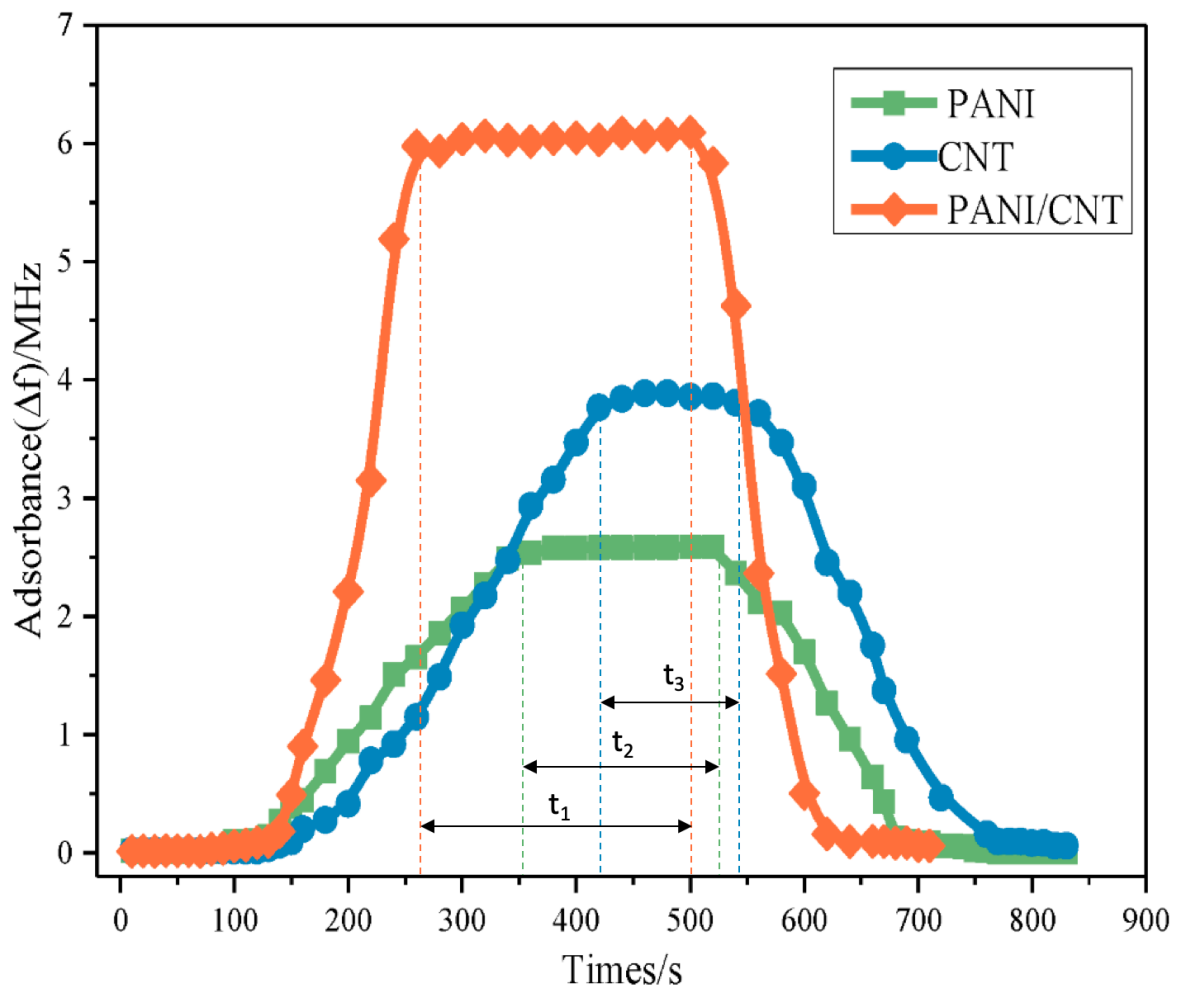
Unsynchronized Response and Recovery Curves [129].

Repeatability is one of the key indicator of sensor performance, including stability baseline, regular response time and regular recovery time, which directly determines whether the sensor can be used in the actual environment instead of in the laboratory stage. Compared with using a single sensor, sensor arrays have worse repeatability, for any un-synchronization of a sensor in arrays may lead to delay and drift problems. As shown in Figure 7, the repeatability of PANI, CNT, and PANI/CNT composite are unsynchronized, which will take unstable data when the acquisition time is not in the public part of t${}_{1}$, t${}_{2}$ and t${}_{3}$ [129].

Nanotechnology [30,130] and biotechnology [131] are often used at the material level to solve unified repetitive problems by speeding up self-recovery and improving stability of the sensing principle. Also, the time-frequency transform is very useful in solving signal delay and drift. Luna et al. [132] present the FFT to eliminate the delay and baseline drift of the sensor array and achieve good results. Xing et al. [133] have developed an FFT-based signal processing algorithm to maintain a fast response of the sensor array with a response time reduction from 10 s to 2 s or less.

The problem of repetitiveness has been discussed repeatedly for many years. More specific solutions can be found in this paper [19].

More discussion here is about the challenge of reusability, which is a trend in the future of smart gas sensors. It has been repeatedly mentioned how to construct a specific gas fingerprint using a weak selectivity gas sensor array. So the reusability of smart gas sensing is based on the same set of sensing systems to identify multiple gases under different application scenario, by replacing the sensor type and pattern recognition algorithm. On the one hand, it is limited by different standards of gas sensors. On the other hand, pattern recognition technology needs model training based on specific training data (means that complex data correction and model training are required for each reuse), the reusability of smart sensing is still not achievable.

In fact, it is almost impossible to achieve a uniform gas sensor specification. A preferred method is to mimic Field-Programmable Gate Array (FPGA) to build an editable smart gas sensor platform. A. Ait Si Ali et al. [134] have tried reusability research on smart gas sensing. They integrate multiple sensors on SOC, including in house fabricated 4 × 4 SnO${}_{2}$ based sensor and seven commercial Figaro sensors, and combine multiple machine learning algorithms to detect more than ten kinds of gases; various sensors can be activated as needed. In addition to the unified platform, another promising approach is to build large-scale open gas datasets [135], which enable Data scientist focus on developing more efficient gas sensing algorithms without complex sensor manufacturing and gas detection experiments. For example, Liu et al. [136] propose a novel data processing method using the bio-inspired neural network modeled on the mammalian olfactory system, which can automatically learn features without cumbersome steps such as denoising, feature extraction, and simplification. When biometrics and computational neuroscience are sufficiently developed, the reusability of smart sensors will surpass various standard limits.

### 4.2. Circuit Integration and Miniaturization

**Figure 8:**
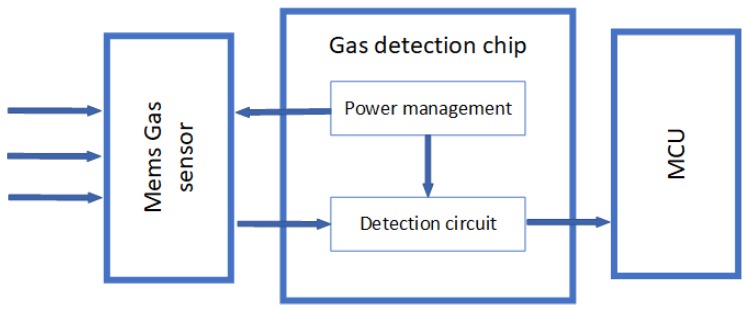
Structure of Gas Detection Systems [140].

Circuit integration is a prerequisite for making energy-efficient and portable/wearable [137,138] gas sensing systems. Such gas detection systems must be self-adjusting and operate without help for any laboratory equipment. Thus, gas sensing systems include specific gas sensors, readout circuits, data processing circuits including high precision analog to digital converters (ADCs), and interface circuits for communicating with Micro-controller Unit(MCU), such as Inter-Integrated Circuit (IIC), Serial Peripheral Interface (SPI), etc [139]. Figure 8 shows a standard structure [140].

The challenges of circuit integration for smart gas sensors include (1) reducing the size of sensor and circuit; (2) avoiding the effects of gas sensing principle between different sensors; (3) resolving signal transmission conflicts in the circuit between sensors; (4) reducing circuit power consumption.

**Figure 9:**
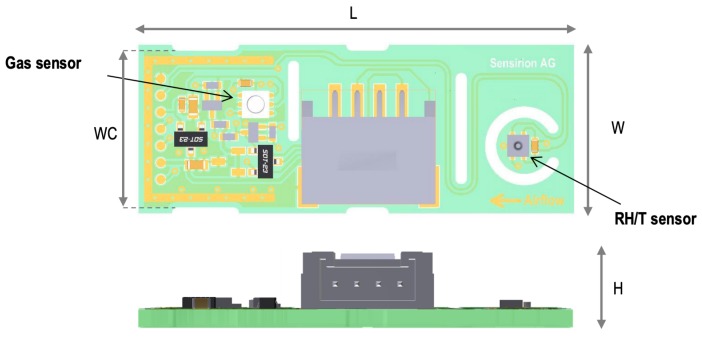
Smart Gas Sensing SOC [141].

The study of SOC provides a solution to the above challenges by highly integrating electronic components, as shown in Figure 9 [141].

Smart gas sensing SOC consists of a Micro-Electro Mechanical Systems (MEMS) gas sensor and a Complementary Metal Oxide Semiconductor (CMOS) integrated circuit [142].

J. Wang et al. [139] have detailedly introduced the fabrication process of the MEMS gas sensor and integrated circuit, and the area of the chip is only 1 × 1.5 mm${}^{2}$. Though there is no power-hungry detector in a MEMS gas sensor, the heating element becomes the limiting factor for power consumption [143]. C. Seok et al. [144] use Capacitive MEMS Ultrasonic Transducer (CMUT) to avoid heating components and successfully reduce the power consumption of the circuit to the microwatt (μW) level. Finally, the potential work in smart gas sensing SOC is the improvement of the detection circuit. The recent related literature was proposed by M. Chen et al. [140]. They removed analog components such as ADCs in integrated circuits, instead only one ring-oscillator is needed to detect the resistance changes of the sensors. The experimental results show that the chip’s resistance measurement range is 1 Ω–500 MΩ, which is equivalent to the output frequency range of 145 Hz to 4.11 MHz and can significantly satisfy the performance of MEMS gas sensors. There is still much work to be done in circuit integration and miniaturization, which requires the joint efforts of materials, electronics, and other fields.

### 4.3. Real-Time Sensing

Real-time sensing is urgently needed for fire detection and industrial production to detect problems in time and protect personnel safety [145]. For example, a smart fire detector should react before smoke breaks out and warn about the substance going to start burning [146]. Unlike portable smart gas sensors, real-time smart gas sensing faces the challenge about calculation time and data transfer, which need to adequately collect gas information from every sensing region and process gas data quickly to analyze the result. A single smart gas sensing SOC can only detect gases in small nearby areas, and the MCU on the SOC does not have enough performance to support fast pattern recognition calculation. So how to build a wireless sensor network (WSN) to deploy multiple gas sensors and get reliable computing power to train a model, are key points for real-time sensing.

**Figure 10:**
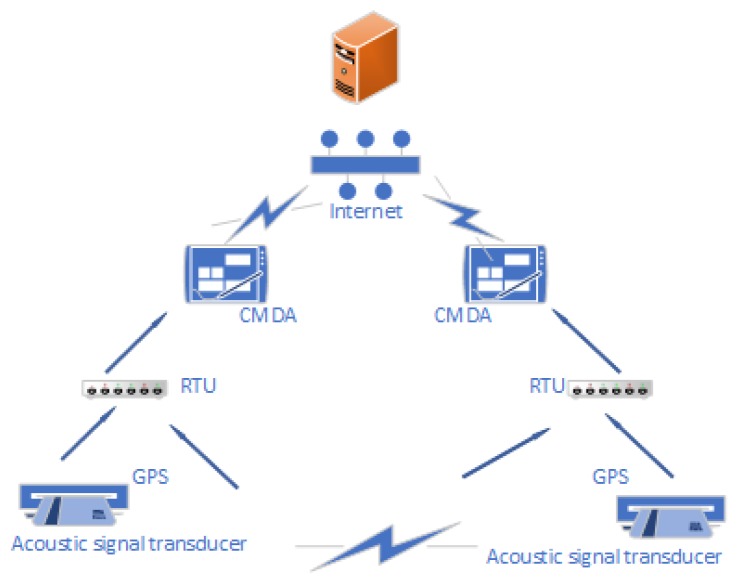
A Structure of Centralized WSN [9].

One solution is to send the data to the cloud server for model calculation, which is called centralized WSN [147]. F. Wang et al. [9] construct acoustic sensing based leak detection system by WSN, as shown in Figure 10. The remote terminal units (RTU) at upstream and downstream synchronously sample the 4–20 mA signals with the Global Positioning System (GPS) timing, and send data to the computing server with Code Division Multiple Access (CDMA) for processing and calculation. The above system only uses WSN with a single-hop structure, which lacks robustness and popularization in complex environments.

**Figure 11:**
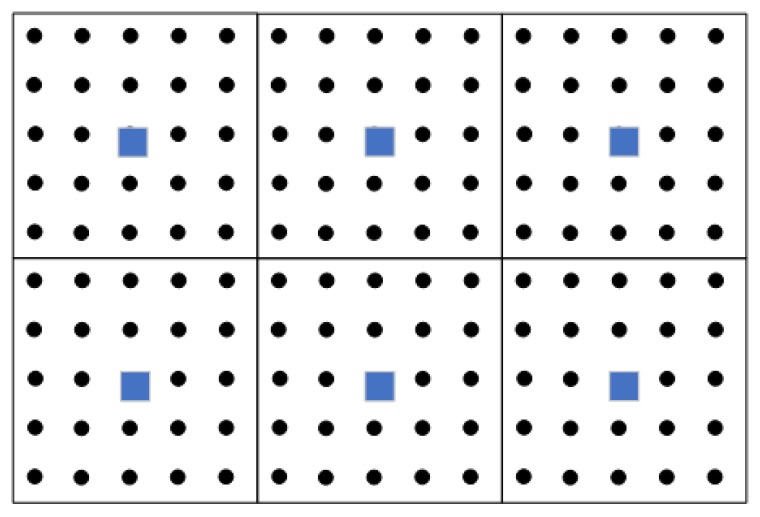
A Distributed WSN Based on Fog Calculation [149].

Another solution is to deploy multiple weak computing nodes locally by WSN according to fog computing, which can save energy and bandwidth consumption, and extend the lifetime and utility of the network [148]. S. Mahfouz et al. [149] proposed a sophisticated framework for the detection and estimation of the parameters of multiple gas sources in WSNs. As shown in Figure 11, the sensing area is parted into some distinct clusters to get rid of transmission impairments and network failures. Each cluster is managed by a cluster head (as the square in Figure 11), that is a smart Central Processing Unit (CPU), responsible of handling (gathering and synchronizing) data, performing calculations, exchanging information with the sensors (as the dot in Figure 11) in a cluster, and communicating with other cluster heads.

For real-time smart gas sensing, further research is needed to optimize sensor deployment locations [150] and reduce communication power consumption [145] in WSNs to ensure accurate, long-term and real-time sensing.

## 5. Conclusions

Smart Gas Sensing is a cross-disciplinary field that includes physical and chemical material sciences, electronic circuits, statistics, chemometrics, communication networks, and machine learning methods. In this paper, we have introduced the general process of smart gas sensing technology for readers, includes sensing materials and sensor array, signal processing for drift compensation and feature extraction, and gas pattern recognition technology based on machine learning. The different technologies at each stage are detailed and compared, including their principles, advantages, disadvantages, and application fields, which aim to provide readers with reference cases in terms of smart gas sensing. Finally, the challenges of intelligent gas sensing are summarized, including reusability, circuit integration, and real-time. Viable solutions are given by related papers.

Sensor arrays combine various sensors to achieve the complementary performance. MOS sensors have long-lasting life and short response time of sensing, but they need to elevate operating temperature, which causes high energy consumption. CPCs and CNTs can work at room temperature, but they are sensitive to humidity, which will cause drifts. QCM/SAW has accurate detection performance for the humidity. For the catalytic sensor, they can complement the detection of flammable gases. The flexible combination of the above sensors into the sensor array can be widely applied to various complex gas detection scenarios based on gas pattern recognition technology. Finally, the application of ubiquitous networks and microelectronics technology accelerates the use of smart gas sensing from the laboratory to specific applications.

The unmanned, precise, and economical advantages of smart gas sensing are attracting the attention of more scientists and companies. In future, brain-like sensing will probably provide a promising direction for smart gas sensing: (1) sensor arrays consist of gas sensitive protein receptor to improve sensitivity and fault tolerance; (2) sensor attention control to simulate neuronal excitation and suppression, for odor contrast enhancement and normalization; (3) brain-like ANN [151] to handle complex relationships between gases and simulating human odors.

## Figures and Tables

**Table 1:** Comparison of Sensitivity Characteristics of Humidity Sensors.

Materials	Measure Range	Sensitivity	Response/Recovery Time	Temperature	Mass/Thickness of Coated Film
SAW with PVA Film	16–72.8%RH	89.34 kHz/%RH	-/-	20 °C	-/910 nm
Patterned on the Reflectors [59]
SAW with PVA Film	15–59.1%RH	23.09 kHz/%RH	-/-	20 °C	-/865 nm
Opened at IDT Pads [59]
Nanoflower TiO${}_{2}$-shaped QCM [60]	0–97%RH	15.3 Hz/%RH	9 s/3 s (At 97%RH)	20 °C	11827 ng/1–4 μm
Nanosphere TiO_2_-shaped QCM [60]	0–97%RH	18.9 Hz/%RH	6 s/3 s (At 97%RH)	20 °C	11801 ng/1–3 μm
Hollow Ball-like TiO_2_-coated QCM [60]	0–97%RH	33.8 Hz/%RH	5 s/2 s (At 97%RH)	20 °C	11676 ng/300 nm
PDDAC /GO, Film-based QCM [61]	0–97%RH	25.4 Hz/%RH	7 s/3 s (At 97%RH)	25 °C	5518 ng/-
Acidized-MWCNTs -coated QCM [62]	11–95%RH	221.4 HZ/%RH	49 s/6 s (At 95%RH)	25 °C	21114 ng/-

**Table 2:** Summary of Gas Sensitive Materials Which Used in Sensors Array.

Material	Advantages	Limiting Factors	Application
	(1) Small size	(1) Poor specificity and selectivity	
	(2) Low cost	(2) High operating temperature	
Oxide Semiconductor	(3) Short response time	(3) Affected by humidity and poisoning	Almost all areas
[26,27,28,29,30,31,32,33]	(4) Long-lasting life	(4) Nonlinearity at high temperature	
	(5) Simple circuit	(5) High energy consumption	
	(1) Strong sensitivity	(1) Long response and recovery time	(1) Biological sensor
Conductive Polymer	(2) General operating temperature	(2) Low selectivity	(2) Disease detection
Composites	(3) Strong biomolecular interactions	(3) High cost	(3) Food quality testing
[34,35,36,37,38,39]	(4) Various preparation processes	(4) Easy affected by humidity	(4) Plating material
	(1) High sensitivity	(1) High cost	(1) Environmental monitoring
	(2) Strong adsorption capacity	(2) Complicated production	(2) Disease detection
Carbon Nano-materials	(3) Sturdy and lightweight	(3) Non-uniform standard	(3) Military field
[40,41,42,43,44,45,46,47,48,49,50,51,52,53,54]	(4) Stable and suitable for mixing	(4) Complex mechanism
	other materials		
	(5) Quick adsorption capacity		
	(1) High sensitivity and short response time	(1) Affected by temperature and humidity	(1) Electronic nose
Acoustic Wave Sensor	(2) Low power consumption	(2) Complex coating process	(2) Environmental monitoring
[30,55,56,57,58,59,60,61,62]	(3) Suitable for almost all gases	(3) Poor signal-to-noise performance	(3) Food quality testing
	(4) Long-term stability		
	(1) Low cost	(1) Catalyst poisoning	(1) Combustible gas detection
Catalytic Sensor	(2) Low sensitivity to humidity	(2) Low sensitivity	(2) Drunk driving detection
[63,64,65,66]	(3) Good reproducibility	(3) Low selectivity	

**Table 3:** Gas sensor arrays reported in Different Literatures [122].

Formaldehyde (ppm)	Ethanol (ppm)	Acetone (ppm)	Touene (ppm)	BP	ELM	SVM
100	0	0	0	W	W	C
0	150	0	0	W	C	C
0	0	200	0	C	C	C
0	0	0	10	C	C	C
10	50	0	0	C	C	C
10	0	200	0	C	C	C
10	0	0	50	C	C	C
50	100	0	0	C	C	C
50	0	10	0	C	C	C
50	0	0	150	C	C	C
100	150	0	0	C	C	C
100	0	50	0	W	C	C
100	0	0	200	C	C	C
10	50	50	50	C	C	C
50	100	50	50	C	C	C
50	50	10	50	C	C	C
100	50	50	50	C	C	C
			Accuracy (%)	82	94	100
			Train Time (s)	17.80	0.04	0.95

**Table 4:** Algorithm comparison.

	Key Point	Advantage	Disadvantage	Filed
		(1) Comprehensible	(1) Sensitive for sample distribution	Increasing the selectivity to gases
		(2) Insensitive to noise	(2) Slow speed for recognition	Identifying similar gases
KNN	k value	(3) Low cost for retraining	(3) High spatial complexity	
[101,102,103,104,105,106,107]	The types of mixed gases data	(4) Good combination with other algorithms	(4) Heavy calculation burden
			(5) Poor interpretability	
		(1) Strong theoretical basis	(1) Sensitive of noise	Improving the accuracy of sensor
		(2) Processing the small sample	(2) Tough choice for kernel function	Small gases sample data
SVM	Kernel function	(3) Good generalization ability	(3) Long learning time	Calibrating sensors
[110,111,112,113,114,115]	The amount of mixed gases data	(4) Resolve non-line questions	(4) Poor application in large samples	
		(5) Solving the optimal solution		
	Weight	(1) Good learning ability	(1) A plenty of parameters requirement	Handling nonlinear relationships
ANN	Activation function	(2) Good parallel processing capability	(2) Poor interpretability for output	Predicting gas interaction
[91,119,120,121,122,123,124,125,126]	No. of hidden layers	(3) Strong compatible for error	(3) Too long learning time	Calibrating sensors
		(4) Resolve complex non-line questions	(4) Easy to overfit	
